# Emerging Species and Genome Editing Tools: Future Prospects in Cyanobacterial Synthetic Biology

**DOI:** 10.3390/microorganisms7100409

**Published:** 2019-09-29

**Authors:** Grant A. R. Gale, Alejandra A. Schiavon Osorio, Lauren A. Mills, Baojun Wang, David J. Lea-Smith, Alistair J. McCormick

**Affiliations:** 1 Institute of Molecular Plant Sciences, School of Biological Sciences, University of Edinburgh, Edinburgh EH9 3BF, UK; grant.gale@ed.ac.uk (G.A.R.G.); alejandra.schiavon@ed.ac.uk (A.A.S.O.); 2 Centre for Synthetic and Systems Biology, University of Edinburgh, Edinburgh EH9 3BF, UK; baojun.wang@ed.ac.uk; 3 Institute of Quantitative Biology, Biochemistry and Biotechnology, School of Biological Sciences, University of Edinburgh, Edinburgh EH9 3FF, UK; 4 School of Biological Sciences, University of East Anglia, Norwich NR4 7TJ, UK; l.mills@uea.ac.uk (L.A.M.); d.lea-smith@uea.ac.uk (D.J.L.-S.)

**Keywords:** CRISPR/Cas, CRISPRi, genetic circuits, genome engineering, inteins, genome-scale models, mutant library, optogenetics, serine integrase, sigma factors, synthetic biology

## Abstract

Recent advances in synthetic biology and an emerging algal biotechnology market have spurred a prolific increase in the availability of molecular tools for cyanobacterial research. Nevertheless, work to date has focused primarily on only a small subset of model species, which arguably limits fundamental discovery and applied research towards wider commercialisation. Here, we review the requirements for uptake of new strains, including several recently characterised fast-growing species and promising non-model species. Furthermore, we discuss the potential applications of new techniques available for transformation, genetic engineering and regulation, including an up-to-date appraisal of current Clustered Regularly Interspaced Short Palindromic Repeats/CRISPR associated protein (CRISPR/Cas) and CRISPR interference (CRISPRi) research in cyanobacteria. We also provide an overview of several exciting molecular tools that could be ported to cyanobacteria for more advanced metabolic engineering approaches (e.g., genetic circuit design). Lastly, we introduce a forthcoming mutant library for the model species *Synechocystis* sp. PCC 6803 that promises to provide a further powerful resource for the cyanobacterial research community.

## 1. Introduction

Cyanobacteria are a diverse phylum of photosynthetic prokaryotes that are found in a wide variety of marine and freshwater habitats [1,2,3,4]. Oxygenic photosynthesis evolved approximately 2.5 billion years ago in the predecessors to modern-day cyanobacteria [5]. Their early success led to a significant increase in free oxygen (O_2_) in the Earth’s atmosphere and the subsequent evolution of most aerobic organisms [6,7,8]. Today, cyanobacteria account for 20%–30% of global carbon dioxide (CO_2_) fixation [9]. 

The ability of cyanobacteria to convert captured carbon into a wide variety of complex organic molecules makes them promising platforms for the sustainable production of biofuels and high-value chemicals [10,11,12]. Compared to plants, cyanobacteria offer several advantages for biotechnological applications, including (1) higher photosynthetic efficiencies [13,14], (2) capacity to grow in hostile living environments (e.g., in extremes of temperature, salinity and pH) [1,3,4], (3) the ability to be cultured on non-arable land with minimal nutrients [15], and (4) the relatively rapid and inexpensive generation of mutants (predominantly in model species) [16]. Moreover, chloroplasts descend from an internalised cyanobacterium [17], thus certain physiological and biochemical features are conserved in eukaryotic photosynthetic organisms, making cyanobacteria excellent chassis for production of plant-derived natural products, such as terpenes [18,19]. Currently, several companies are investigating the use of cyanobacteria for producing biochemicals, including biofuels (Algenol), inks (Living Ink Technologies) and pigments for food and cosmetics (DIC Corp., Lumen Bioscience, ScotBio). Nevertheless, several hurdles still need to be overcome for more widespread industrial adoption.

One key issue has been the relatively slow growth rates of cyanobacterial species, including those developed as models such as *Synechocystis* sp. PCC 6803 (PCC 6803) and *Synechococcus elongatus* PCC 7942 (PCC 7942). For example, PCC 6803 has a doubling-time of *ca*. 7 h under standard growth conditions [20], compared to 20 min for *Escherichia coli* and 2 h for *Saccharomyces cerevisiae* [21,22]. Recently, several genetically tractable cyanobacterial species have been characterised with faster growth rates that are comparable with *S. cerevisiae* [20,23,24]. In this review, we will discuss these strains and other non-model species that demonstrate the potential for cyanobacteria to close the gap between industrially viable heterotrophic and phototrophic bio-platforms or are useful organisms for investigating unique aspects of cyanobacterial biology and how they adapt to different environments. 

A further significant challenge limitation has been the limited availability of molecular tools to engineer cyanobacteria. However, the past few years have seen a rapid proliferation of characterised tools and parts for cyanobacteria, including CRISPR/Cas-based systems [25,26,27,28]. This has driven the widespread adoption of the synthetic biology paradigm for the design of biological tools based on the bottom-up approach of recombining standardised parts or modules (e.g., promoters, ribosome binding sites (RBS), coding sequences and terminators) [29,30,31,32,33,34,35]. Many of those tools were initially developed in *E. coli* or *S. cerevisiae* and have been adapted and modified for use in cyanobacteria [36,37]. A significant drawback is that they may behave differently in cyanobacteria and between different cyanobacterial species. Thus, the functionality of all new tools ported for cyanobacterial applications must first be validated [35,38]. Here, we will outline several recent developments in genome engineering using CRISPR/Cas and recombinase approaches, as well as the opportunities and limitation of recent tools developed in *E. coli* that show promise for cyanobacterial research and biotechnology applications. 

To date, 290 draft genomes and 85 full genomes are available online in the CyanoBase database (http://genome.microbedb.jp/cyanobase [39]). The growing availability of cyanobacterial genome sequencing data has helped to foster the development of genome-scale models (GSMs) for a variety of species, ranging from model species (e.g., PCC 6803) to the industrial relevant strain *Arthrospira* (Spirulina) *platensis* NIES-39 [40,41,42,43]. As with *E. coli* [44], GSMs have allowed cyanobacterial researchers to adopt a systems biology approach to propose and predict the outcomes of engineering strategies. Here, we will also highlight examples where GSMs have successfully guided efforts to modify metabolism to improve production in cyanobacteria and how GSMs could drive an increase in available cyanobacterial omics data.

Finally, we will introduce a forthcoming barcoded mutant library in PCC 6803 called CyanoSource. CyanoSource will be a powerful tool to develop a deeper understanding of metabolism in PCC 6803, guide work in other species (including new fast-growing strains) and will set a benchmark for high-throughput research in the cyanobacterial field.

## 2. “Non-Model” Species: Requirements for Uptake and Genetic Manipulation 

Cyanobacterial research has focused primarily on model organisms that are straightforward to culture under laboratory conditions, amenable to genetic modification and can be frozen for long-term storage [16,45,46,47]. The uptake of more recalcitrant, non-model species for laboratory research can present significant challenges. Table 1 outlines some basic features that are desirable for culturing and engineering. Below, we have highlighted several new or known “non-model” cyanobacteria, including their potential benefits, challenges for widespread uptake and industrial usage, and where applicable, progress towards efficient genetic manipulation (Table 2).

### 2.1. The Emergence of Fast-Growing and Stress-Tolerant Synechococcus Strains 

Cyanobacterial strains that can achieve growth rates comparable with heterotrophic microbes could be of significant value to basic research and the biotechnology industry. Recently, three new *Synechococcus* strains have been reported with high growth rates: *Synechococcus elongatus* UTEX 2973 (UTEX 2973), *Synechococcus elongatus* PCC 11801 (PCC 11801) and *Synechococcus* sp. PCC 11901 (PCC 11901). UTEX 2973 was first described in 2015 as a fast-growing, stress-tolerant strain that was re-isolated from a previously characterised fast-growing strain that had lost the ability for fast growth [20,55]. Under high light (>500 µmol photons m^−2^ s^−1^) and high temperatures (38–42 °C), UTEX 2973 can achieve doubling times similar to that of *S. cerevisiae* (*ca*. 2 h) during the early growth phase [20,24,56]. Under those conditions, UTEX 2973 can produce biomass twice as fast as its close relative PCC 7942, despite only a small number of nucleotide differences between the two genomes [57]. However, growth was not examined for longer periods of time (i.e., >24 h). At lower temperatures (30 °C), growth of UTEX 2973 is slower than PCC 7942 or PCC 6803 at 300 or 500 µmol photons m^−2^ s^−1^ [35], and slower than 6803 when cultured for longer periods (up to 10 days) under high light (750 µmol photons m^−2^ s^−1^) [24]. UTEX 2973 is not naturally competent, but is amenable to conjugation and genome editing by CRISPR/Cas [20,58]. Furthermore, naturally transformable mutants of UTEX 2973 have been described [33], while three point mutations in PCC 7942 can reportedly lead to growth rates similar to that of UTEX 2973 [57]. The latter finding could have a significant impact on cyanobacterial research as PCC 7942 is a naturally transformable and widely used model species. 

PCC 11801 was isolated from India and first described in 2018 [23]. Similarly to UTEX 2973, PCC 11801 is tolerant to high light and temperatures and exhibits fast growth rates (i.e., a doubling time of 2.3 h). Furthermore, PCC 11801 is tolerant of high levels of NaCl (i.e., it can grow at sea salt concentrations (*ca*. 0.7 M), whereas PCC 7942 cannot) and it is naturally transformable. The genome sequence of PCC 11801 is highly similar to PCC 7942 and UTEX 2973 (*ca*. 83%). Thus, existing plasmid vectors used for engineering PCC 7942 are widely compatible in PCC 11801.

One of the best-performing strains reported to date is PCC 11901, which was isolated in Singapore from an estuarine environment enriched with nitrogen and phosphorous compounds [24]. PCC 11901 is tolerant to high light levels, achieves fast growth rates comparable to UTEX 2973, and is tolerant to a wide range of salinities, similar to PCC 11801. Although UTEX 2973 grows faster than PCC 11901 during the early growth phase, PCC 11901 reportedly outperforms UTEX 2973 when grown for longer time periods (i.e., >24 h). PCC 11901 accumulated 2–3 times more biomass when grown alongside PCC 6803, PCC 7942, *Synechococcus* sp. PCC 7002 (PCC 7002) and UTEX 2973, achieving an OD_730_ = 101 and a biomass of 18.3 g dry weight per litre. PCC 11901 is naturally transformable with efficiencies similar to that of PCC 7002, as demonstrated by the generation of markerless mutants using methods previously described in PCC 7002 [59]. 

### 2.2. Nostoc punctiforme ATCC 29133 

*Nostoc punctiforme* ATCC 29133 (ATCC 29133) is a nitrogen (N_2_)-fixing, heterocyst-forming cyanobacterium which forms a symbiotic relationship within the coralloid roots of plants. ATCC 29133 is a useful organism for investigating symbiotic relationships between plants and N_2_-fixing cyanobacteria [60]. It has also been key to understanding the biosynthetic pathway of scytonemin, a natural sunscreen against UV damage produced by many cyanobacterial species [61]. Transformation of ATCC 29133 was first described in 1994 [62] and has since been further developed to generate scytonemin-deficient mutants [63]. Mutants were obtained via conjugation, either by random transposon insertion into the open reading frame of the scytonemin biosynthesis operon or by allelic exchange. ATCC 29133 appears amenable to selection using chloramphenicol, neomycin, streptomycin and *sacB* markers. 

### 2.3. Cyanothece sp.

*Cyanothece* species are a group of unicellular cyanobacteria that can perform photosynthesis and fix N_2_ within the same cell via temporal separation of the two processes. N_2_ fixation occurs during the dark period when O_2_ levels are low. They are natural contributors to N_2_ fixation in rice paddies, and, therefore, could play an important role in reducing agricultural fertiliser use [64,65]. A genetic transformation protocol for producing targeted gene knockouts in *Cyanothece* sp. PCC 7822 was developed in 2010 [66], where a single-stranded DNA fragment encoding a spectinomycin resistance cassette was electroporated into cells, leading to integration of the cassette at random points in the genome via non-homologous recombination. Despite testing *Cyanothece* sp. ATCC 51142, PCC 7424, PCC 7425, PCC 8801 and PCC 8802, mutants could only be generated in PCC 7822 via this technique. More recently, Liberton et al. [67] reported a method for generating targeted mutations in *Cyanothece* sp. ATCC 51142 using triparental mating. A plasmid encoding two methylases was required in order to make the cargo plasmid more resistant to digestion. Using this system, a kanamycin resistance cassette was inserted into a targeted chromosomal site.

### 2.4. Arthrospira sp.

*Arthrospira* species are the source of high-value nutraceuticals (e.g., Spirulina) and natural blue pigments in food (e.g., the phycobiliprotein, *C*-phycocyanin) [68,69]. They tolerate high levels of alkalinity and can be cultured in a variety of closed or open (e.g., race way pond) environments. Nevertheless, *Arthrospira* sp. are highly resistant to genetic modification due to an abundance of native restriction–modification systems that can rapidly degrade heterologous DNA [70]. The most efficient transformation system reported to date used a Tn5 transposase expression cassette to generate random integration events in the genome of *Arthrospira platensis* C1 and selection via a spectinomycin resistance cassette [53]. DNA degradation was minimised by encapsulation in liposomes and mixing with a type 1 restriction inhibitor prior to electroporation. Transformed cells resistant to spectinomycin were reportedly stable for several months. It is tempting to speculate that delivery of a CRISPR/Cas system with this approach could enable targeted genome editing in *Arthrospira* sp. 

### 2.5. Leptolyngbya sp. 

*Leptolyngbya* species are widely distributed in terrestrial and freshwater environments [71], and are, therefore, of great ecological interest. *Leptolyngbya* sp. BL0902 is also used for the production of biomass and bioproducts, as it can grow at a range of industrially viable temperatures, tolerate high salt concentrations, pH extremes and variable light conditions. Growth rates in the laboratory and in outdoor ponds are similar to those of *Arthrospira* species [72]. Conjugal transformation of *Leptolyngbya* sp. BL0902 (BL0902) with broad host range vectors based on RSF1010 was successfully carried out in 2012 [72,73], although two antibiotics were required to limit the appearance of spontaneous resistant mutants. Conjugation was used for generation of a transposon library and introduction of an expression plasmid.

### 2.6. Fremyella diplosiphon

*Fremyella diplosiphon* is a filamentous, heterocyst-forming, freshwater species that can adjust its photosynthetic receptors and antenna to differences in light intensity and quality. Detailed methods for genetic manipulation are available that allow for the generation of unmarked mutants [74]. First, the plasmid of interest is methylated to protect it from digestion in *F. diplosiphon*. The plasmid is then introduced via triparental mating and transconjugates selected on plates containing neomycin [75]. Unmarked mutants can then be generated using *sacB*.

### 2.7. Marine Synechococcus sp. and Prochlorococcus sp.

Marine *Synechococcus* and *Prochlorococcus* genera are responsible for approximately a quarter of ocean primary productivity [2], and are, therefore, of great academic interest. Genetic manipulation of *Prochlorococcus* sp. (including the introduction of a heterologous plasmid) has not been reported. However, *Prochlorococcus* strains can be cultured on semi-solid agar plates in the presence of specific ‘helper’ heterotrophic bacteria [76], fulfilling the initial requirement for genetic manipulation. Gene deletion has been reported in the marine *Synechococcus* species WH7803, WH8102 and WH8103 [77,78]. Plasmids were introduced into cells via biparental mating or electroporation. Transformants were plated on semi-solid (0.3% *w*/*v*) agar plates and kanamycin was used as the selectable marker. Via this method, self-replicating and suicide plasmids were introduced, which facilitated targeted mutations.

### 2.8. Thermosynechococcus elongatus 

*Thermosynechococcus elongatus* BP-1 is a thermophilic cyanobacterium with optimal growth at 55 °C, making it ideal for biotechnology applications that require high temperatures [79]. *T. elongatus* BP-1 proteins are also ideal for purification and crystallographic studies, due to their increased stability at high temperatures [80]. A natural transformation method for chromosomal integration has been developed for *T. elongatus* BP-1, with either kanamycin or chloramphenicol used for selection of transformants [81]. More recently, *T. elongatus* PKUAC-SCTE542 has been highlighted as a naturally transformable strain with high growth rates that is sensitive to spectinomycin [82].

### 2.9. Chlorogloeopsis fritschii and Fischerella muscicola 

*Chlorogloeopsis fristchii* sp. PCC 6912 and *Fischerella muscicola* PCC 7414 are two of the most complex species of cyanobacteria, in that they are filamentous, heterocyst-forming strains able to undergo multiplanar cell division and thereby create multiseriate filaments [83]. Introduction of expression plasmids via conjugation and biolistic DNA transfer methods has been reported in both species. Conjugation was made possible by the partial removal of the exopolysaccharide sheath by introducing a salt washing step. 

### 2.10. Chroococcidiopsis thermalis 

*Chroococcidiopsis thermalis* is found in environments with extremes of temperature (both hot and cold) [84]. Furthermore, these extremophile cyanobacteria can survive long periods of desiccation and high levels of solar radiation that few other organisms can tolerate. *C. thermalis* incorporates chlorophyll *f* in its photosystems, allowing absorption of far-red light not available to other photosynthetic organisms [85]. *Chroococcidiopsis* species have been suggested as possible candidates for terraforming other planets [86]. Expression plasmids have been introduced into several strains of *C. thermalis* via conjugation [84]. 

### 2.11. Gloeobacter violaceus PCC 7421

*Gloeobacter violaceus* PCC 7421 (PCC 7421) is a primordial cyanobacterium that lacks thylakoid membranes [87]. PCC 7421 localises proteins involved in photosynthesis and respiration to specific regions of the cytoplasmic membrane [88]. Despite its very slow growth, a method of transforming PCC 7421 with an expression vector via conjugation has been developed [89]. 

**Table 2:** Summary of genetic manipulations carried out in model and non-model cyanobacterial species discussed in Section 2.

Species	Strain	Desirable Features from Table 1	Transformation Method	Reported Selection Markers	Agar Medium	References
*Synechococcus* sp.	UTEX 2973	1), 2), 3), 4), 5), 6)	Conjugation CRISPR/Cas *pilN* mutants are naturally transformable	Apramycin Chloramphenicol Kanamycin Spectinomycin Streptomycin	BG-11	[20,33,35,58]
	PCC 11801	1), 2), 3), 5), 6)	Naturally transformable	Spectinomycin	BG-11	[23]
	PCC 11901	1), 2), 3), 5), 6)	Naturally transformable	Acrylic acid Spectinomycin	AD7	[24]
*Nostoc punctiforme*	ATCC 29133	1), 2), 3), 4), 5), 6)	Conjugation	Chloramphenicol Neomycin *sacB* markers Streptomycin	Allen and Arnon	[62,63,90]
*Cyanothece* sp.	PCC 7822	1), 2), 3), 5)	Electroporation	Spectinomycin	BG-11	[66]
	ATCC 51142	1), 2), 3), 4), 6)	Conjugation	Kanamycin	BG-11 ASP2	[67]
*Arthrospira platensis*	C1	1), 2), 3), 4), 5)	Electroporation	Spectinomycin	Zarrouk	[53]
*Leptolyngbya* sp.	BL0902	1), 2), 3), 4), 5), 6)	Conjugation	Chloramphenicol Erythromycin Neomycin Spectinomycin Streptomycin	BG-11	[72,73]
*Fremyella diplosiphon*	SF33	1), 2), 3), 4), 6)	Conjugation	Kanamycin Neomycin *sacB* markers	BG-11/HEPES	[74,75,91]
Marine *Synechococcus* sp.	WH7803 WH8102 WH8103	1), 2), 3), 6)	Conjugation Electroporation	Kanamycin	SN	[76,77,92]
Marine *Prochlorococcus* sp.		1), 3)	-	Spectinomycin	Pro99	[76,93]
*Thermosynechococcus elongatus*	BP-1	1), 2), 3), 4), 5), 6)	Naturally transformable	Chloramphenicol Kanamycin	BG-11	[81]
	PKUAC-SCTE542	1), 2), 3), 6)	Naturally transformable	Spectinomycn	BG-11	[82]
*Chlorogloeopsis fritschii*	PCC 6912	1), 2), 3), 4), 5)	Biolistic Conjugation	Kanamycin Neomycin	Allen and Arnon	[83,90]
*Fischerella muscicola*	PCC 7414	1), 2), 3), 4), 5)	Biolistic Conjugation	Kanamycin Neomycin	Allen and Arnon	[83,90]
*Chroococcidiopsis thermalis*		1), 2), 3), 4), 5)	Conjugation	Neomycin	BG-11	[84]
*Gloeobacter violaceus*	PCC 7421	1), 2), 3), 4), 5)	Conjugation	Streptomycin	BG-11	[89]

## 3. Current and Future Strategies for Genome Engineering in Cyanobacteria

### 3.1. CRISPR/Cas Genome Editing in Cyanobacteria 

The RNA-guided CRISPR/Cas family of enzymes has been the driving force for a revolutionary step change in precision genome editing capacity in almost every field of biology, including photosynthetic biology [94,95,96]. Briefly, all CRISPR/Cas genome editing systems exploit the Class II family of CRISPR-associated endonuclease (Cas) enzymes (comprising types II, V and VI Cas) [97,98]. The type II-A Cas of *Streptococcus pyogenes* (SpCas9) was first demonstrated as a site-specific RNA-guided DNA cleavage tool by Jinek et al. [99]. Since then, a vast array of CRISPR/Cas technologies have been produced and continue to be developed at a rapid pace [100,101,102,103,104,105]. Genome editing studies using CRISPR/Cas have now been reported in several cyanobacterial species, including PCC 6803, PCC 7942, UTEX 2973 and the filamentous strain *Nostoc* (*Anabaena*) PCC 7120, from four separate labs (for recent reviews, see [25,27,106]). 

**Figure 1:**
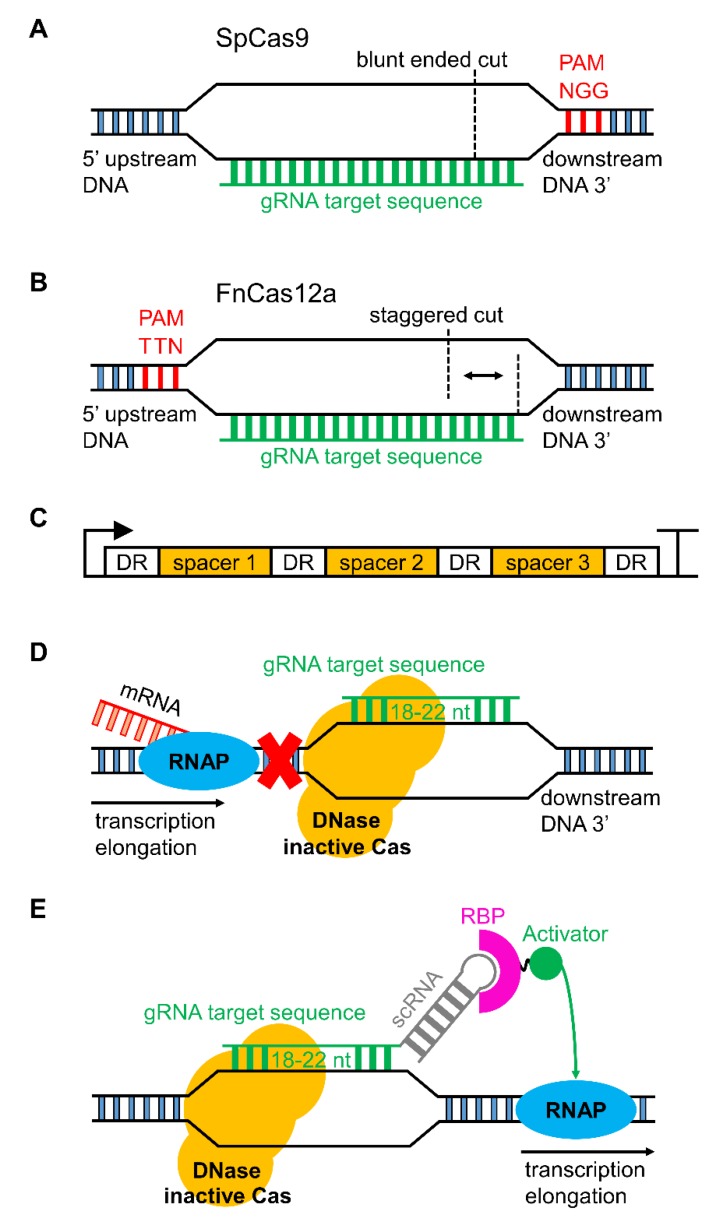
Basic overview of CRISPR/Cas-based genome editing and CRISPR interference in cyanobacteria. (**A**) The type II-A CRISPR/Cas system comprising SpCas9 that is targeted by a guide RNA (gRNA, green) upstream of an NGG protospacer adjacent motif (PAM) (red). SpCas9 generates a blunt-ended double-stranded break (DSB) in DNA (black dashed line). (**B**) The type II-V CRISPR/Cas system comprising FnCas12a that is targeted by a gRNA downstream of a TTN PAM. Cas12a confers a staggered DSB in DNA that results in a five-nucleotide overhang. (**C**) Illustration of a single expression cassette containing three spacers (i.e., a spacer array, orange) that are flanked by direct repeat regions (DRs, white). Cas12a recognises and cleaves spacers in response to a DR to generate a mature gRNA from each spacer. (**D**) Overview of transcriptional inhibition by CRISPRi. DNase inactive Cas enzymes (e.g., dCas9 or ddCas12a) are targeted to a DNA locus by a gRNA (typical length indicated as 18–22 nucleotides), which blocks RNA polymerase (RNAP, blue) to prevent mRNA (red) synthesis. (**E**) Example of transcriptional activation by CRISPRa. DNase inactive Cas enzymes (e.g., dCas9 or ddCas12a) are targeted by a gRNA fused to a scaffold RNA (scRNA, grey). The scRNA recruits an RNA binding protein (RBP, pink) fused to a transcriptional activator (green) (e.g., [108]), which subsequently leads to activation of RNAP and transcription elongation.

We will not cover the specific mechanisms of all the available CRISPR/Cas tools here (for reviews, see [94,96]). We will focus on Cas9, which confers a blunt-ended double-stranded break (DSB) in DNA (Figure 1A), and type V-A Cas (Cas12a, or previously Cpf1), which produces a staggered DSB (Figure 1B) [107]. Use of CRISPR/Cas systems for gene editing relies on a synthetic single-guide RNA (sgRNA or gRNA), which, for Cas9, is a fusion of a crRNA (CRISPR-RNA) and a tracrRNA (trans-activating crRNA). For CRISPR/Cas9 systems, gRNAs are commonly expressed from a DNA template, with each gRNA transcribed from a single expression cassette. The crRNA component is customised for targeting a specific genomic locus and the tracrRNA acts as a scaffold for recruitment of Cas9. In contrast, Cas12a enzymes possess an intrinsic RNase activity that facilitates autoprocessing of gRNAs that can be expressed from ‘spacer arrays’ (Figure 1C) and a tracrRNA fusion is not required. Spacer arrays are comprised of spacers that code for gRNAs, which are each separated by a direct repeat (DR). The DR facilitates recognition and cleavage of the precursor RNA by Cas12a to form mature gRNAs. To date, all Cas isoforms that target DNA require a 2–6 nucleotide sequence called a protospacer-adjacent motif (PAM) site for Cas to bind DNA and generate a DSB. Depending on the type of Cas used, PAM sites are situated immediately upstream or downstream of the gRNA target locus. As PAM sites are sequence-specific, the choice of Cas enzyme used can impact on the gRNA loci available.

In cyanobacteria (as in other prokaryotes that lack an endogenous non-homologous end joining (NHEJ) pathway) [109,110]), CRISPR/Cas has been used as an enhancement tool to improve the frequency of targeted mutation by HR [58,111,112,113]. In brief, expression of Cas and a gRNA mediates cleavage at a specific DNA target site. The co-expressed editing template contains homology flanks (*ca*. 1 kb in length) and subsequent repair modifies the locus and mutates the PAM site to avoid repeated cleavage. The potential advantages of CRISPR/Cas over established HR strategies in model cyanobacteria such as PCC 6803 [16] are that 1) a markerless mutation is induced at the DNA target site in a single event; 2) multiple sites could be modified simultaneously, provided the appropriate gRNAs and editing templates are co-expressed; and 3) CRISPR/Cas systems could be more efficient for engineering species that are not naturally transformable (e.g., by conjugation or electroporation). Potential drawbacks include toxicity of the Cas enzyme and the time required to cure new mutants of the CRISPR/Cas vector following editing. One approach to accelerate the latter issue is the inclusion of a negative selection marker (e.g., *sacB*) on the CRISPR/Cas vector [113]. Thus far, SpCas9 is the only Cas9 reported to have been expressed in cyanobacterial strains, including PCC 6803, PCC 7942 and UTEX 2973 (Table 3) [58,111,112]. Expression of SpCas9 has been linked to toxicity and failure to recover colonies following transformation or conjugation in all three species, even at low expression levels. It remains unclear why SpCas9 appears toxic in cyanobacteria, or whether other Cas9 enzymes may be more compatible.

In contrast to Cas9, expression of Cas12a in the cyanobacterial strains examined so far does not appear to result in toxicity [57,113,114]. Gene editing using Cas12a from *Francisella novicida* (FnCas12a) has been demonstrated in PCC 6803, PCC 7942, UTEX 2973 and *Nostoc* PCC 7120. An additional advantage of Cas12a is that the intrinsic RNase activity allows for the design of a single expression cassette containing multiple gRNAs to target several DNA loci simultaneously (Figure 1C) [115,116,117]. A recent multiplexing study with Cas12a designed an array of 25 gRNAs on a single plasmid that simultaneously edited multiple genomic target sites in mammalian cells [105]. 

Previously, reports of gene editing with FnCas12a in cyanobacteria have been limited to a single lab [57,114]. However, Niu et al. [113] have recently also demonstrated that *Nostoc* PCC 7120 can be edited by FnCas12a. A high efficacy was demonstrated for FnCas12a-mediated gene editing of single target sites (83%) by attempting to generate knockout mutants in 26 different genes using 52 gRNAs (two per gene) [113]. In addition, a markerless double-knockout mutant was generated for two genes required for heterocyst formation (*hetR* (alr2339) and *hetN* (alr5358)) in a single step following co-conjugation with two CRISPR/FnCas12a editing vectors carrying different antibiotic selection markers. Lastly, a conditional knockout was generated for the essential gene *polA* (alr1254; DNA polymerase I) by replacing the RBS of the *polA* promoter with a theophylline-induced riboswitch [118,119]. Successfully transconjugated lines were only viable when grown with theophylline. Transconjugates were then cured of the self-replicating CRISPR/FnCas12a vector by withdrawing antibiotics and using *sacB* counter selection on sucrose plates. 

Thus far, FnCas12a is the only reported Cas12a used in cyanobacteria genome editing studies. One general constraint of CRISPR/Cas editing is the specificity of the PAM site required for DNA cleavage. Of potential interest to cyanobacterial researchers is the growing availability of different Cas12a isoforms (e.g., AsCas12a and LbCas12a from *Acidaminococcus* sp. and *Lachnospiraceae bacterium*, respectively) and those engineered to recognise alternative PAM sequences (Table 4) [96,120]. Increased flexibility in PAM recognition will allow for more choice when targeting loci for genome editing [120,121]. New Cas enzymes continue to be identified but remain to be evaluated in cyanobacteria, such as CasX, which also produces a staggered DSB but is smaller than Cas12a [122]. If CasX is less toxic than Cas9, it may provide a useful new set of tools for genome-editing in cyanobacterial species. 

### 3.2. Serine Integrases for Generating Multiple Knock-ins

The most commonly used methods for genome engineering in cyanobacteria still rely on HR and the use of selective markers. For example, to generate a gene knockout or a knock-in mutant, heterologous DNA must be integrated with a selective marker (e.g., an antibiotic resistance cassette). Thus, the ability to generate mutants in a given species with multiple insertional mutations is limited by the availability and efficacy of selective markers. To overcome this limitation, several methods have been developed for generating markerless mutants, which allows mutant strains to undergo further genetic modifications. One of the most widely used markerless techniques in PCC 6803 uses a two-step HR approach with the negative selection marker *sacB*, which produces levansucrase, an enzyme conferring sensitivity to sucrose [16]. Nevertheless, generating a fully segregated markerless mutant for a single locus takes *ca*. 4 weeks to several months, depending on the target locus, while sequential engineering for multi-mutant strains can be very time consuming. Tsujimoto et al. [123] demonstrated an insertion of 20.8 kb in PCC 6803, but this was achieved by a laborious five-step HR process using *ca*. 4 kb at a time. Therefore, new methods need to be developed to allow large DNA insertions and/or multiple loci engineering in a more efficient timeframe. 

**Figure 2:**
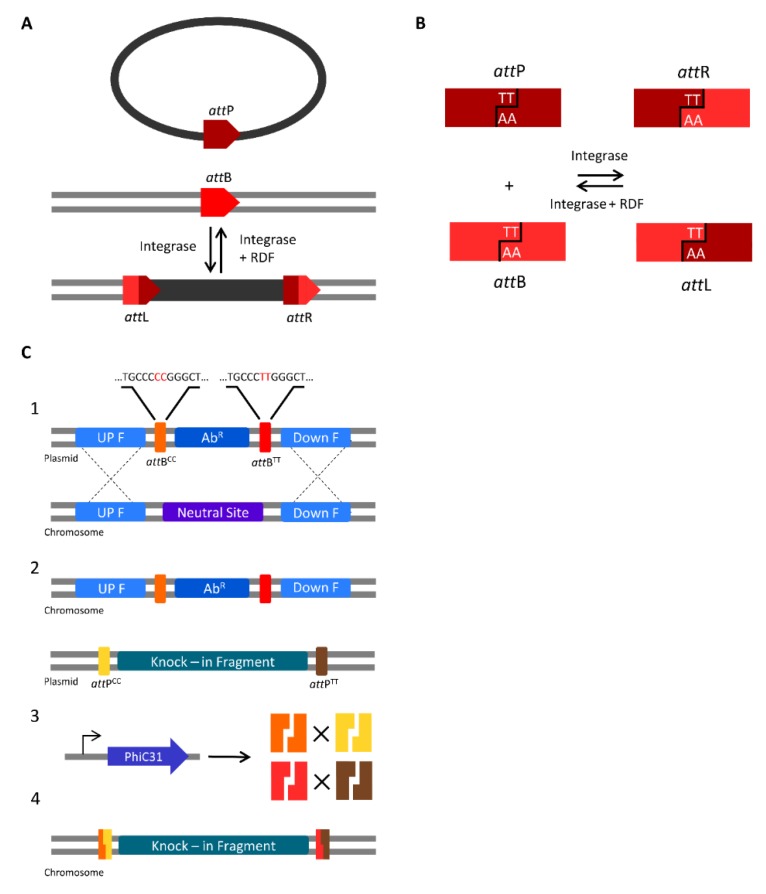
Mechanism of action of serine integrases and knock-in strategies. (**A**) DNA rearrangement with serine integrases: *att*B and *att*P sites recombine to make *att*L and *att*R sites. In the presence of the cognate recombination directionality factor (RDF), the reaction is reversed [124]. (**B**) The serine integrases bind to the *att*P and *att*B sites, generate a staggered 2 bp cut to produce two halves at each site, and then rotate to swap the two halves from each site. If the halves are complementary, relegation occurs to generate *att*L and *att*R sites. (**C**) A serine integrase-mediated knock-in strategy for cyanobacteria. Integration of a landing pad (the landing pad shown carries orthogonal *att*B^CC^ and *att*B^TT^ sites) occurs by homologous recombination (HR) at a neutral site in the cyanobacterial chromosome (C1). A self-replicating vector carrying the donor DNA fragment flanked by *att*P^CC^ and *att*P^TT^ sites and a PhiC31 integrase expression cassette is introduced into the cyanobacterial strain (C2). Expression of PhiC31 integrase results in the recombination of the orthogonal *att*B and *att*P pairs (C3), replacing the “landing pad” with the donor DNA to generate an unmarked knock-in mutant (C4).

Serine integrases are a subfamily of the site-specific recombinases that catalyse DNA rearrangement through small DNA sequences (<50 bp) called attachments (*att*) (commonly used for GateWay cloning) [124]. Serine integrases catalyse recombination between the *att* sites from linear or circular DNA that can result in excision, integration or inversion of DNA sequences, depending on the position and orientation of the *att* sites. Temperate bacteriophages encode serine integrases to catalyse integration of their DNA into bacterial genomes through recombination of *att*P (phage) and *att*B (bacteria) attachment sites, generating *att*L (left) and *att*R (right) sites (Figure 2A). Serine integrases bind to the *att*P and *att*B sites to make a staggered cut in the central region, generating halves with a two base-pair 3′ overhang. Then, rotation takes place to swap the *att*P and *att*B half-sites and finally, the two bp complementary overhangs religate to generate *att*R and *att*L sites that cannot recombine again unless a recombination directionality factor (RDF) is present (Figure 2B). Six pairs of nonpalindromic central overlap sequences (TT, CT, GT, CA, CC, and TC) can be used to create orthogonal sites and allows multiple *att* sites to be used simultaneously (e.g., *att*P^TT^ will only recombine with *att*B^TT^ in a specific orientation) [125]. The control of directionality and orthogonality have made serine integrases attractive tools for genome engineering and genetic logic gate design [126,127]. In contrast to CRISPR/Cas-based approaches, site-specific recombination using serine integrases does not rely on endogenous DNA repair pathways, such as NHEJ or HR. Although CRISPR/Cas knock-in approaches are able to generate small insertions in a single step, the size of the insertion remains limited by the efficiency of HR. In addition, unlike Cas9, no toxicity has been reported with the usage of serine integrases in several organisms [124,126,128].

Serine integrases have been used for genome engineering in a variety of organisms, including mice [129], *Drosophila melanogaster* [130], *S. cerevisiae, E. coli* [126] and *Clostridium ljungdahlii* [128]. A recent strategy outlined a portable method to simplify the introduction of multiple genomic insertions using the orthogonal *att* sites of the PhiC31 serine integrase [126]. Firstly, one or more selective markers flanked by two orthogonal *att*B sites were integrated into the genome of *E. coli* as “landing pads”. Although Snoeck et al. [126] used HR to introduce the landing pads, other techniques such as CRISPR/Cas have been used for *att* site integration [128]. Secondly, a donor vector carrying an expression cassette for PhiC31 integrase and the DNA fragment(s) to be inserted flanked by *att*P orthogonal sites was introduced to generate insertions specific for each corresponding landing pad by *att*B × *att*P recombination (Figure 2C). 

Using a single landing pad, a 10.3 kb DNA fragment was inserted with 100% efficiency. Simultaneous recombination with three landing pads generated a triple-knock-in mutant (all fragments were *ca*. 2.5 kb in size), with an efficiency of 75%. 

In cyanobacteria, integration of one or more landing pads at a given locus could proceed either by a two-step HR approach if the species is naturally transformable [16], or by CRISPR/Cas-mediated HR [114]. CRISPR/Cas could also be used to insert landing pads at multiple loci in a single step [113], Broad host range vectors able to self-replicate in cyanobacteria (e.g., pPMQAK1) could be used to construct the donor vector [35]. To generate a markerless knock-in mutant following serine intergrase-mediated recombination, the donor vector could be cured from the strain by inclusion of the negative selection marker *sacB* on the vector backbone. Thus, serine integrases could emerge as a useful tool for the generation of multi-knock-in markerless mutants for large DNA fragments in cyanobacteria. Generating a library of strains with *att* “landing pads” in combination with high-throughput assembly methods, such as Golden Gate MoClo for the assembly of the donor vectors [35], could significantly speed up the design-build-test cycle. 

## 4. Known and Novel Tools for Regulating Gene Expression in Cyanobacteria

### 4.1. Gene Regulation with CRISPRi and Synthetic Small Regulatory RNAs

The emergence of CRISPR interference (CRISPRi) and synthetic small regulatory RNA (sRNA) tools in cyanobacterial research has allowed for fine modulation of gene expression at both the transcriptional and translational levels. For translational repression, sRNA tools employ an antisense RNA to bind a specific mRNA transcript target and generate an RNA duplex [131,132]. The RNA duplex suppresses translation and subsequently targets the mRNA transcript for degradation. sRNA-based approaches have been demonstrated in PCC 6803 and PCC 7002, including a paired termini antisense RNA approach and an Hfq-mediated system using sRNAs fused to a MicC scaffold [132,133,134]. Both approaches demonstrated up to 90% reduction in protein expression. 

For transcriptional repression, CRISPRi approaches make use of DNase-inactive variants of Cas, called “dead” Cas9 (dCas9) or DNase “dead” Cas12a (ddCas12a; also known as ddCpf1) (Figure 1D) [100,117]. To date, SpdCas9 is the only reported DNase-inactive Cas used in cyanobacterial research, but it has been used successfully to repress gene expression in PCC 6803, PCC 7002, PCC 7942 and *Nostoc* PCC 7120 [35,133,135,136,137,138]. Unlike SpCas9, SpdCas9 does not appear to be toxic. However, in our lab, we have observed a reduction in growth rates in PCC 6803 when SpdCas9 was expressed at high levels, suggesting that low/medium strength promoters should be used when designing SpdCas9 expression cassettes. Decreased growth rates and changes in cell morphology and division have been observed in *E. coli* when expressing SpdCas9 at high levels [139].

Multiplexing of gRNAs to target several genes simultaneously with SpdCas9 has been demonstrated in PCC 6803 [137,140]. Kaczmarzyk et al. [137] demonstrated simultaneous repression of six native genes. However, a potential limitation to multiplexing using dCas9 is the need for individual expression cassettes for each gRNA. Kaczmarzyk et al. [137] reported issues with vector recombination in PCC 6803 due to repeated use of common promoters and terminators for each gRNA cassette. ddCas12a may provide an improvement over SpdCas9 in cyanobacteria for multiplexing gRNAs and repression of multiple loci, as demonstrated in *E. coli* [117]. Cas12a requires a DR length of 19 base pair (bp) to generate mature gRNAs from spacer arrays [141], which is significantly less than that of most promoters and terminators; this may help to reduce plasmid vector size requirements and mitigate recombination issues when multiplexing is required. However, cloning spacer arrays can be challenging due to the multiple repeated sequences, and is not always achievable even using commercial DNA synthesis companies. 

CRISPRi using SpdCas9 has been reported to reduce protein expression of heterologous reporter genes (e.g., YFP) between 40% and 99%, depending on the gRNA(s) used [35,140]. Native gene repression has been shown to vary, with a maximum reduction of 94% achieved for *glgC* in PCC 7942 [136,140]. Yao et al. [136,140] targeted a range of native genes with CRISPRi in PCC 6803 and observed reductions of <90% for *slr0942*, *sll0990*, and *slr1192*, but more modest knock down of 50% for *slr0091*. Thus, validation of gRNA(s) is important to ensure effective transcriptional repression, which can be time consuming. To achieve more robust and consistent down regulation, it may be advantageous to combine CRISPRi and sRNA to simultaneously modulate transcription and translation. Furthermore, CRISPR/Cas variants have been characterised that target RNA, and thus also can modulate expression at the translational level. For example, Cas13 cleaves single-stranded RNA [142], and in its deactivated form (dCas13), can bind mRNA and suppress translation [143]. Cas13 may provide additional tools for RNA manipulation and additional strategies for gene and multigene repression studies.

Finally, dCas9 has also been used to drive gene expression by so called CRISPR activation (CRISPRa). CRISPRa relies on gRNA(s) modified to include an extended hairpin sequence (termed a scaffold RNA, scRNA) that function, for example, to recruit an RNA binding protein (RBP) fused to a transcriptional activator [108]. Thus, when the dCas9-scRNA complex binds to a target locus, the scRNA recruits the appropriate machinery to drive transcription (Figure 1E). As the dCas9 is unmodified, CRISPRi and CRISPRa could be achieved concurrently with the expression of multiple gRNAs and scRNAs for simultaneous gene repression and activation [108,144]. In *E. coli*, effective gene activation by CRISPRa does require appropriately positioned PAM sites situated at specific locations upstream of the transcription start site. Recent work shows that shifting the gRNA target site by as little as two nucleotides can lead to a significant loss in activation [145]. Nevertheless, these approaches could improve on current strategies in cyanobacteria to both express heterologous pathways and repress native gene expression for the redirection of metabolic flux towards desired products [137]. 

### 4.2. Sigma Factors and RNA Polymerase as Regulatory Tools for Gene Transcription 

The highlighted serine integrase recombination and CRISPR-based approaches are examples of promising tools for genome engineering and gene regulation in cyanobacteria. However, applying these approaches often requires careful regulation of the composite parts by inducible and, ideally, orthogonal gene expression systems to generate predictable outputs [146,147,148]. 

Sigma factors are critical components required for transcription initiation in bacteria that interact with the core RNA polymerase (RNAP) complex to facilitate binding to specific DNA promoter regions [149]. Thus, different sigma factors are involved in driving the transcription of different subsets of genes, and are themselves expressed by different environmental or stress inputs [150,151]. All cyanobacterial sigma factors belong to the sigma 70 family [152], although several others exist in bacteria [153]. 

Recent reports have provided good evidence that sigma factors may be compatible with RNAP complexes from different bacterial species, paving the way for a potential novel orthogonal expression system in cyanobacteria. *Nostoc* PCC 7120 contains twelve sigma factors that regulate gene expression according to environmental conditions [154]. In a recent study, Wells et al. [155] tested the sigma factors from *Nostoc* PCC 7120 in *E. coli* and observed that several cyanobacterial promoters were able to drive transcription in *E. coli* only when sigma factors from *Nostoc* PCC 7120 were co-expressed. Similarly, sigma factors from *Bacillus subtilis* can be co-expressed in *E. coli* to construct an orthogonal expression system [156]. Thus, testing non-sigma 70 factors in cyanobacteria may help to identify novel tools for orthogonal transcriptional regulation [157]. For example, *E. coli* have a sigma 54 factor [158,159], which if functional in cyanobacteria, could allow for transcription from sigma 54-dependant promoters as a novel orthogonal trans-acting expression system [152]. Liu et al. [160] have recently demonstrated in *E. coli* that sigma 54-dependent promoters can be combined with CRISPRa and have a higher dynamic range compared to sigma 70-dependent promoters. 

Similarly, heterologous RNAP systems could be employed. For example, the T7 RNAP is a single subunit RNA polymerase of viral origin that is commonly used as an expression tool in *E. coli* due to its orthogonality to bacterial transcription machinery [161,162,163]. Use of T7 RNAP has also recently been demonstrated in PCC 6803 and PCC 7942 [164,165]. As in *E. coli*, native sigma factors in cyanobacteria do not interact with the T7 promoter sequence (P_T7_), so genes driven by P_T7_ are transcribed only if the cognate T7 RNAP is expressed. Directed evolution approaches have produced new variants of T7 RNAP with altered promoter recognition characteristics [161,166]. P_T7_ has also undergone substantial analysis, with Komura et al. [167] testing transcriptional activity of 7847 P_T7_ variants. T7 RNAP has also been adapted to act as a photoactivatable genetic switch in *E. coli* with dark-off/light-on properties [168]. The latter system could be of use in cyanobacterial biotechnology; for example, light- or dark-dependent control of protein production (e.g., for light-sensitive bioproducts) with a variety of different promoter strengths. 

### 4.3. The Potential of Optogenetic Systems 

Several small-molecule inducible/repressible systems have been characterised in *E. coli* [169,170,171]. However, only a small number have been characterised in cyanobacteria, and thus far, only in model species [31,37,164,172,173,174]. The commonly used lac operon induction system, which uses isopropyl β–d-1 thiogalactopyranoside (IPTG), has been shown to be leaky and have low induction levels, possibly due to the limited capacity of IPTG to diffuse into cyanobacterial cells [119,175,176]. Metal ion inducible promoters have been tested in several cyanobacterial strains [177,178,179], but these are sometimes not practical as many metals ions are present in standard growth media (e.g., BG11 [180]) and toxicity can be an issue. More recently, an arabinose inducible and a rhamnose inducible promoter were characterised in PCC 6803, which showed tight regulation, linear response and sustained expression [31,37]. However, the relatively low availability of inducible/repressible systems in cyanobacterial species compared to model heterotrophs still limits the progress and development of more advanced synthetic circuits for dynamic control of cellular behaviour [108,181]. 

In the last decade, optogenetics (i.e., light-controlled regulation) has emerged as a promising tool for tuning synthetic circuits in mammalian and bacterial cell systems [182,183,184,185,186]. Compared with chemical induction systems, optogenetic systems allow for more targeted, rapid and precise control of genetic elements with increased spatial and temporal control, while being minimally invasive and reversible [187,188,189,190]. Optogenetic systems can be classified broadly into two-component systems (TCSs) or one-component systems (OCSs). 

**Figure 3:**
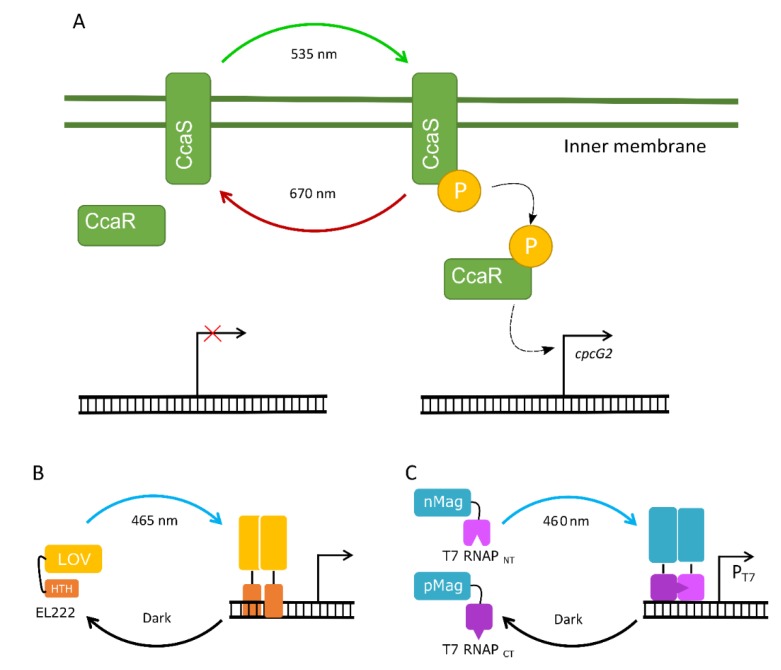
Examples of optogenetic two-component and one-component systems. (**A**) The two-component green light-inducible CcaS/CcaR system native to *Synechocystis* sp. PCC 6803. In the presence of green light (535 nm), the histidine kinase CcaS is phosphorylated and, in turn, phosphorylates the response activator CcaR, which results in expression of cpcG2 [191,196]. (**B**) The EL222 transcription factor from *Erythrobacter litoralis* HTCC2594 is a one-component system. Blue light induces a conformational change between the LOV and the Helix-Turn-Helix (HTH) DNA binding domain, allowing dimerisation and DNA binding [187]. (**C**) Split T7 RNA polymerase (RNAP) fused to the Vivid (VVD) photoreceptor from *Neurospora crassa* is a one-component system. In blue light, the two subunits of VVD (nMag and pMag) interact to assemble the split T7 RNAP (T7 RNAP_NT_ and T7 RNAP_CT_) as a functional RNA polymerase [204].

An optogenetic TCS requires two elements: a light-sensing module and a light-responsive module that is activated by the light-sensing module. For example, the green light-inducible CcaS/CcaR TCS in PCC 6803 relies on the membrane-bound histidine kinase CcaS (i.e., the light-sensing module), which is phosphorylated in green light (Figure 3A) [191]. Phosphorylated CcaS subsequently phosphorylates the cytosolic response regulator protein CcaR (i.e., the light-responsive module) that, in turn, activates the expression of the phycobilisome linker gene *cpcG2*. TCSs have been identified in plants [192], bacteria [191] and fungi that specifically sense UV [193], blue [194,195], green [196,197,198], red [199] or near-infrared light [200]. In contrast, optogenetic OCSs act as both the sensing and responsive modules and are found in the cytosol. So far, only the blue light-activated OCSs that belong to the LOV (Light–Oxygen–Voltage) family of proteins have been characterised in heterotrophic bacteria [187,201], *S. cerevisiae* [186,202] and *Arabidopsis thaliana* [203] (Figure 3B,C). Both systems have been used in the control of genetic circuits at multiple levels, such as transcription and protein activity.

Apart from the green light-inducible CcaS/CcaR system, only one other optogenetic TCS has been characterised in PCC 6803, the near-UV activated UirS/UirR system that regulates phototaxis [205]. The latter has not yet been exploited in cyanobacterial research, but the CcaS/CcaR system has been used in PCC 6803 to regulate GFP expression [196], and drive expression of two T4 phage-derived lysis genes to generate a green light-induced cell membrane lytic system [197]. The CcaS/CcaR system has also been used to modulate GFP in a marine species where the CcaS/CcaR system is absent (*Synechococcus* sp. NKBG 15041c), and demonstrated tight repression under red light and a 20-fold induction of GFP under green light [206]. Thus, cyanobacterial TCSs show promise as tools for transcriptional control in species where those TCSs are not present. 

There are also several optogenetic systems characterised in *E. coli* that could be used in cyanobacterial research. For example, the near-infrared (760 nm) TCS BphP1/PpsR2 from *Rhodopseudomonas palustris* showed rapid response dynamics and a 2.5-fold dynamic range [200]. Recently, an OCS based on the Vivid (VVD) photoreceptor from the filamentous fungus *Neurospora crassa* was used to generate a blue-light-inducible T7 RNAP system (Figure 3B) [204]. In the dark, only low levels of gene expression were observed, while high levels of expression were achieved in blue light (460 nm), with an inducible range of >300-fold. Optogenetic systems responsive to different light wavelengths can also be combined to achieve multichromatic gene control [199]. For example, a red–green–blue (RGB) system was constructed in *E. coli* for production of three different pigments to generate colour biophotographs [188]. The RGB system used a fragmented T7 RNAP that could bind to specific promotors depending on the light input, and demonstrated little crosstalk, high dynamic range and fast responses when induced. 

Currently, the main challenge for porting optogenetic systems into cyanobacteria is ensuring compatibility with native light-responsive components. Cyanobacteria naturally produce many of the cofactors required for light-sensing and light-responsive modules [207], which could provide an advantage when porting heterologous optogenetic systems. However, growing cyanobacteria in specific wavelengths (e.g., blue, green, orange, red) will affect photosynthetic efficiencies and growth [208]. Therefore, optimising optogenetic systems might require testing several sources of light to limit any impacts on photosynthesis and achieve the desired outputs.

### 4.4. Using Inteins to Progress Genetic Circuit Research in Cyanobacteria

Genetic circuits occur in nature and form the basis by which living cells respond and adapt to the surrounding environment [146]. A key goal in synthetic biology is the generation of synthetic genetic circuits that operate as Boolean logic functions to give digital-like control over gene expression in response to environmental stimuli [146,209,210]. Basic Boolean logic functions include ‘AND’ and ‘NOT’ gates: AND gates will give an output signal if all inputs are ‘ON’, while NOT gates will give an output signal only if all inputs are ‘OFF’ [147,211,212]. To date, relatively few synthetic genetic circuits have been constructed in cyanobacteria [213]. These include an oxygen-responsive AND gate in PCC 6803 [214] and four NOT gate variants in PCC 6803, PCC 7942, *Nostoc* PCC 7120, *Synechocystis* sp. WHSyn, and *Leptolyngbya* sp. BL0902 [73]. Currently, two key constraints for making more complex synthetic genetic circuits in cyanobacteria are the relatively small number of characterised inducible expression systems available, and the limited means to integrate multiple input signals (i.e., from different inducible systems) into a single output [215]. Thus, new tools (such as those highlighted in this review) will be required. 

**Figure 4:**
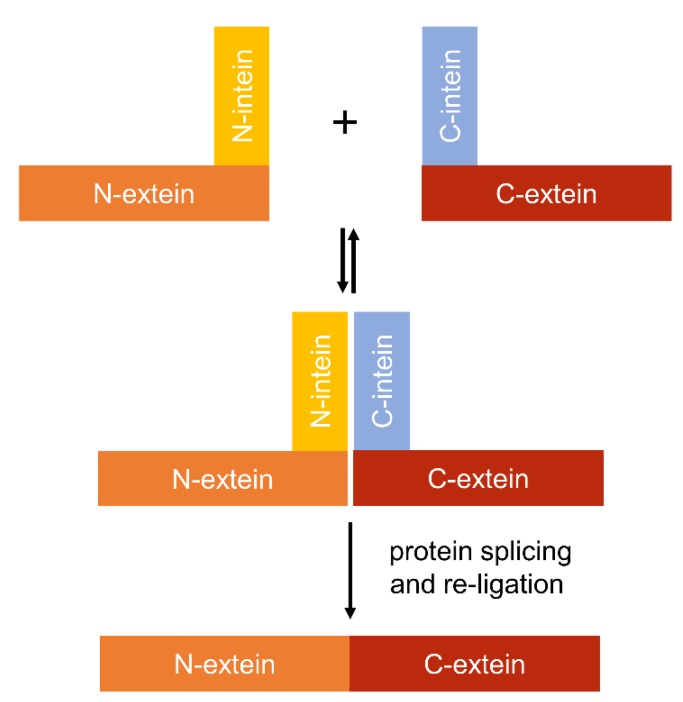
Overview of protein trans-splicing with split inteins. Each precursor protein is composed of a split intein (an *N*-intein half or a *C*-intein half) fused to an extein (an *N*-extein half and a *C*-extein half). When the split inteins are brought into close proximity, they undergo an autocatalytic trans-splicing reaction. During this process, each split intein fragment is cleaved and the extein halves are spliced together to generate an intact protein via a covalent bond.

Inteins are naturally occurring polypeptides (100–150 amino acids) within a larger precursor protein that can excise themselves spontaneously from flanking protein regions (exteins (external proteins)) [216]. Inteins have been identified in several cyanobacteria [217], and a variety of other organisms, including other bacterial species, archaea, viruses and eukaryotes [218,219]. Intein excision involves the cleavage of two peptide bonds, resulting in the formation of a new peptide bond, which ligates the flanking exteins together, forming a newly rearranged protein. This auto-catalytic posttranslational modification is referred to as protein splicing and it exists in two forms, cis- and trans-splicing [220]. In the former, the intein coding sequence is embedded in frame with the gene, such that the precursor protein is produced from a single mRNA transcript and translated as two exteins flanking the intein. Upon cis-splicing, the intein is excised from the precursor protein and the flanking exteins are ligated seamlessly to form a new protein. In contrast, trans-splicing events are facilitated by two precursor proteins translated from separate mRNAs, where each encodes a “split intein” fragment (i.e., an *N*-intein half and a *C*-intein half) fused to an extein (Figure 4). When the precursor proteins are brought into close proximity, the split inteins re-assemble non-covalently [221]. Trans-splicing then occurs, resulting in excision of the split inteins and ligation of the exteins via a covalent bond to generate an intact protein [221,222]. 

Split inteins have been used in *E. coli* to construct a functional two input transcriptional AND gate system using a split T7 RNAP approach [222]. T7 RNAP was separated into two domains, each fused to an *N*- or a *C*-intein half, and expressed from two different inducible promoters. Schaerli et al. [222] showed that expression of both T7 RNAP domain-intein fusions was required to reconstitute a functional T7 RNAP and drive transcription of GFP from P_T7_. Using a similar trans-splicing approach, split inteins have also been used to facilitate re-assembly of split variants of the more complex multidomain transcriptional regulator TetR [223]. Like T7 RNAP, TetR repressible systems have been used in PCC 6803 and PCC 7942 [164]. Thus, these split intein-based gate systems may be straightforward to port into cyanobacteria, provided that the inteins used are orthogonal (i.e., not of cyanobacterial origin) [224]. Split intein strategies could also be used for other transcription factors successfully trailed in cyanobacterial species (e.g., LacI, AraC and RhaS [37,176,214,225]) to construct larger synthetic genetic circuits.

## 5. Genome-Scale Models 

Manipulating cyanobacteria for biotechnology applications is dependent not just on developing better genetic tools but also improving our understanding of cellular metabolism. Genome-scale models (GSMs) are large-scale simulation tools that comprise a global description of the metabolic reactions and pathways of an organism using stoichiometric coefficients and mass balances of the participating metabolites [226]. GSMs are receiving increasing interest due to their predictive power in metabolome and flux changes, thereby making them a valuable tool in metabolic engineering approaches and optimisation for enhanced production of target metabolites [227,228].

Currently, 290 draft genomes and 85 full genomes are available for cyanobacterial species [39]. However, GSMs have only been developed for a small number of these, primarily models such as PCC 6803 [42], PCC 7942 [41] and more recently, UTEX 2973 [43] (Table 5). GSMs from model species can be used as a scaffold to draft GSMs for other cyanobacterial species, as many pathways are conserved [229]. Generating robust and accurate GSMs is an iterative and bottom-up process dependent on the expansion and updating of draft models with available experimental data. Thus, GSM models are continually being improved due to the growing availability of sequencing and omics information. 

Recent cyanobacterial GSMs have attempted to include algorithms to model components of the photosynthetic electron transport chain [41,230,232,235]. However, representing the mechanisms of light capture and electron flow in stoichiometric coefficients is challenging as fluxes cannot be determined experimentally by standard methods (e.g., ^13^C metabolic flux analysis) [41,237,238]. The model iJB785 for PCC 7942 incorporates the impact of light availability on metabolic flux [41]. When used to estimate whether genes were essential or non-essential for survival, the model achieved an accuracy of 78% based on previous experimental data [239]. Recently, the PCC 7942 model was updated (i.e., iJB792) to include whole-cell light absorbance and the rate of photosynthetic O_2_ evolution to predict metabolic reaction fluxes [232]. The updated model demonstrated a 98% correlation between simulated and experimental metabolic fluxes under low- (60 μmol photons m^−2^·s^−1^) and high-light (600 μmol photons m^−2^·s^−1^) conditions. Similarly, the model iSynCJ816 for PCC 6803 can account for changes in energy absorption for different light qualities [230], and achieved a 77% accuracy when compared with experimental results from online databases and literature searches. In comparison, the latest GSM for *E. coli* (iML1515) can predict gene essentiality in minimal media with 16 different carbon sources with an average accuracy of 94% [44]. The relatively higher accuracy of iML1515 relies on the integration of biochemical, physiological, localisation, genetic, transcriptomic, proteomic and fluxomic data. Currently, protein localisation and transcriptomic data are not included in GSMs for cyanobacteria. 

Cyanobacterial GSMs have been used to detect key metabolic differences between species [240] and to identify bottlenecks for the biosynthesis of relevant metabolites [241]. These include a composite GSM for the closely related strains, PCC 7942 and UTEX 2973, based on the model inSyn617 for PCC 6803. The resulting model (iSyu683) identified pathways where resources were allocated differently between PCC 7942 and UTEX 2973, the most prominent difference being carbon uptake rates [233]. An improved model for UTEX 2973 (imSyu593) was recently developed using transient ^13^C-labeling. The flux elucidation revealed nearly complete conversion (>96%) of the assimilated carbon into biomass compared with only 86% conversion in PCC 6803. Comparison of the UTEX 2973 flux map with that of PCC 6803 revealed differences in the synthesis of key Calvin cycle metabolites, fructose-6-phosphate and sedoheptulose-7-phosphate, and production of amino acids, glycine and serine, from the photorespiratory salvage pathway [43]. 

GSMs can be used in cyanobacteria to improve bioproduction. Recently, the model iBJ792 for PCC 7942 was used to determine an optimal solution *in silico* to maximise the production of 2,3-butanediol [232]. GSMs can be also be combined with other computational tools for production optimisation. For example, OptFlux and OptForce are software tools that identify all possible engineering interventions by determining what genes to knock out, or which reaction fluxes in a model need to increase, decrease or fall to zero, to overproduce specific metabolites [242,243]. OptFlux has been combined with GSMs in PCC 6803 to improve the production of *n*-butanol [138]. OptFlux confirmed that genes targeted for manipulation based on previous experimental data were essential for growth. Subsequent repression of the gene targets arrested growth and redirected carbon flux towards the production of *n*-butanol, resulting in a 5-fold yield increase compared to the non-repressed strain. Similarly, OptForce was used to increase the production of limonene [244]. Overexpressing three predicted gene targets (two involved in the pentose phosphate pathway and geranyl diphosphate synthase) resulted in a 2.3-fold improvement in limonene production in vivo. 

Further improvements in the accuracy of GSMs in cyanobacteria are constrained by the lack of available omics data. For example, a large percentage of the predicted proteins in most cyanobacterial genomes are still annotated as unknown or hypothetical [245]. In addition, transcriptomic data and transcriptional regulatory mechanisms are not integrated in current cyanobacterial GSM models, unlike those for other species such as *E. coli* (iML1515) [44,246]. Lastly, incorporation of more accurate models for photosynthesis, light-harvesting and electron transport is required [232]. The current expansion of high-throughput omics technologies and automated cloning facilities (e.g., genome foundries) can be used to generate large amounts of experimental data under different growth conditions, which promises to help overcome current limitations for cyanobacterial GSMs.

## 6. Development of CyanoSource: A Barcoded Mutant Library for *Synechocystis* sp. PCC 6803

Mutant generation is a key tool in bacterial research and for altering species for biotechnology applications. Individual research laboratories currently generate mutants of interest via a variety of different experimental methods, using a range of plasmid systems in sub-strains that can vary significantly at both the phenotypic and genotypic level (e.g., in PCC 6803 [247,248,249]). This raises the issue of whether studies are directly comparable, a key concern given the growing emphasis on reproducibility within the scientific community. The construction of mutant libraries is a consistent and efficient method for the study of protein properties, regulation and function. Mutant libraries have been generated for model photosynthetic eukaryotes, such as *Chlamydomonas reinhardtii* [250] and *Arabidopsis thaliana* [251], and model microbial species including *E. coli* [252] and *S. cerevisiae* [253]. The existence of mutant libraries accelerates the pace of research, avoids unnecessary replication between research groups and helps to improve experimental designs. Targeted mutant libraries also provide knowledge on the essential gene set required for survival, further avoiding wasteful laboratory replication [239]. In addition, many research groups, particularly in developing countries, lack the expertise and resources to generate cyanobacterial mutants.

Cyanobacteria remain underdeveloped for fundamental research and viable biotechnological exploitation. Nevertheless, cyanobacterial research is a rapidly growing field with approximately 13,000 new papers published in the last 5 years, making it the third most studied group of autotrophs behind plants and algae. In PCC 6803, only *ca*. 1050 coding sequences (~30%) have assigned functions, compared to ~80% in *E. coli* and *S. cerevisiae* [226]. Of these, only a small proportion have been characterised in a cyanobacterium [39]. The majority have been assigned functions based on studies of homologues in other bacteria, even though the function and importance of characterised genes may differ significantly between phototrophic and heterotrophic bacteria. Transposon libraries have been reported for two model cyanobacterial species [239,254], but these suffer from common issues associated with transposon mutagenesis, including random large insertions, pleiotropic effects, incomplete saturation of the genome and difficulty in recovering individual mutants of interest. A publicly available genome-wide collection of gene knockout mutants would generate a much-needed step change in resource availability and significantly accelerate research in functional genomics and cellular processes in cyanobacteria. It would also supplement existing algal and plant resources, allowing researchers to further examine genes conserved across the photosynthetic lineage.

**Figure 5:**
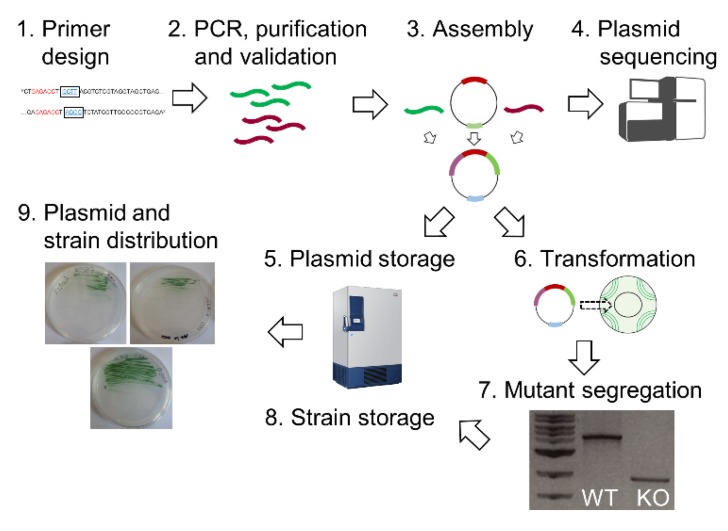
Workflow for the automated production of the CyanoSource resource at two UK DNA Foundry facilities. Primers (**1**) will be used to generate amplicons carrying BsaI sites (red) and appropriate four-nucleotide overhangs (blue, boxed) for MoClo assembly. PCR products will then be purified and validated (**2**) and used to assemble suicide vectors (**3**) for natural transformation of *Synechocystis* sp. PCC 6803. Vectors will be sequenced by MiSeq (**4**) and glycerol stocks made for long-term storage (**5**). PCC 6803 mutants will be generated (**6**), followed by several rounds of re-streaking to produce segregated mutants that will be confirmed by colony PCR (**7**), and stored as glycerol stocks (**8**). For distribution, plasmid stocks are planned to be supplied by Addgene and mutants supplied by the University of East Anglia CyanoSource Hub as streaked plates from glycerol stocks (**9**).

Gene characterisation studies remain challenging and time-consuming but recent developments in automation [255] can streamline and shorten the process of one of the major experimental hurdles: generation of targeted mutants in which the gene of interest has been deleted. DNA assembly and bacterial transformation are complex manual tasks that are time consuming and can suffer from high error rates. Investment by the UK Research and Innovation councils in automated DNA Foundry technologies has provided an opportunity to overcome these challenges [255]. In collaboration with the Earlham DNA Foundry (EDF) and the Edinburgh Genome Foundry (EGF) we will carry out the generation of the plasmids and mutants for this library by using a recently established MoClo system for cyanobacteria [35]. This resource, termed CyanoSource, will target 3456 genes in PCC 6803 (Figure 5). Conditional mutants (i.e., specialised mutants that require an external stimulus to repress a gene) will be constructed for essential genes that cannot be removed. Here, where appropriate, we will use a copper-sensitive promoter that switches off the gene when copper is present [256,257,258]. For the cyanobacterial community, a genome-wide library of mutants and genetic parts generated via automation will guarantee a standard of quality control not otherwise achievable. 

In addition to automation, this library will be barcoded to allow for the tracking of individual strains within a pooled mutant library. This powerful approach has been described in *S. cerevisiae* [253] and *C. reinhardtii* [250], where barcoded libraries can be subjected to different conditions and the relative fitness of individual mutants in a population can be determined via deep sequencing. Construction of this library will begin in November 2019. All plasmids and mutants will be made available to UK and international researchers via a public database, which will be updated throughout the project.

## 7. Conclusions

Given the ongoing advances in complex synthetic biology tools to finely modulate metabolism in microbes, the future looks bright for progressing both fundamental and applied cyanobacterial research. The topics outlined in this review highlight just some of the current exciting methods that could be used to generate a step change for cyanobacteria researchers by improving transformation efficiencies, gene regulation and capacity for metabolic engineering. Development of the techniques and resources outlined in this review should significantly improve our knowledge of this environmentally important phylum, especially for poorly characterised species. Moreover, their application towards the development of cyanobacteria as a renewable biotechnology platform could have immense implications, not just commercially but also in replacing polluting fossil fuels usage for chemical production and reducing carbon emissions.

## Figures and Tables

**Table 1:** Desirable features required for culturing and genetically engineering cyanobacterial strains.

(1) Capacity to grow on agar plates and generate isolated colonies. (2) Amenability to heterologous DNA uptake, either naturally using native DNA import systems [33,48], or via conjugation (i.e., tri- or bi-parental mating) or electroporation. (3) Sensitivity to antibiotics for selection following DNA uptake [49]. (4) Lack of native endonucleases that digest heterologous DNA. If present, the efficiency of DNA uptake can be improved by selecting for strains where endonucleases have been inactivated [48]. Otherwise specific methylases, restriction inhibitors and liposomes could be employed during delivery [50,51,52,53]. (5) Ability to take up broad-host-range self-replicating plasmids (e.g., RSF1010-based) for heterologous gene expression. (6) Capacity for genomic integration via allelic exchange (e.g., homologous recombination (HR) to facilitate the generation of gene knockouts or genomic integration of gene expression cassettes. Ideally, species will be amenable to the generation of unmarked mutants, which is important for industrial applications. Unmarked mutants can be generated using negative selection markers (e.g., *sacB*) [16,54] or by CRISPR/Cas [25].

**Table 3:** Cyanobacterial species where CRISPR/Cas has been used for gene editing.

Species and Strain	Cas Type	Expression System	Reference
*Synechococcus elongatus* PCC 7942	SpCas9	episomal	[111]
*Synechocystis* sp. PCC 6803	FnCas12a	episomal	[114]
*Synechococcus elongatus* UTEX 2973			
*Nostoc* sp. PCC 7120			
*Synechococcus elongatus* UTEX 2973	SpCas9	episomal	[58]
*Synechocystis* sp. PCC 6803	SpCas9	chromosomal	[112]
*Synechococcus elongatus* PCC 7942	FnCas12a	episomal	[57]
*Synechococcus elongatus* UTEX 2973			
*Nostoc* sp. PCC 7120	FnCas12a	episomal	[113]

**Table 4:** The available PAM sequences for cyanobacterial species highlighted in this study for different Cas variants. Only species with full genomes available are shown. The Cas variants indicated are *Streptococcus pyogenes* Cas9 (SpCas9), *Francisella novicida* Cas12a (FnCas12a), *Acidaminococcus* sp. Cas12a (AcCas12a, and variants AsCas12a-RR and ASCas12a-RVR), *Lachnospiraceae bacterium* Cas12a (LbCas12a) and *Deltaproteobacteria bacterium* CasX (CasX). The average number of PAM sites (in brackets) per kb of genome are shown (N = A, T, C, G; V = A, C, G; Y = C, T). Genome data was sourced from The European Nucleotide Archive (https://www.ebi.ac.uk/ena) and The National Centre for Biotechnology Information (https://www.ncbi.nlm.nih.gov/genome).

Cyanobacteria	Genome Size (bp)	SpCas9 (NGG)	FnCas12a (TTN)	AsCas12a and LbCas12a (TTTV)	AsCas12a-RR (TYCV)	ASCas12a-RVR (TATV)	CasX (TTCN)
*Arthrospira platensis* C1	6,089,210	134	168	24	46	21	33
*Arthospira plantensis NIES 39*	6,788,435	114	171	37	46	22	33
*Chroococcidiopsis thermalis PCC 7203*	6,315,792	89	175	38	41	17	34
*Cyanothece* sp. ATCC 51142	4,934,271	118	221	35	40	23	39
*Cyanothece* sp. PCC 7822	6,091,620	114	210	33	39	21	36
*Gleobacter violaceus PCC 7421*	4,659,019	170	89	17	42	6	26
*Nostoc punctiforme strain ATCC 29133*	8,234,322	114	194	43	40	20	35
*Nostoc* sp. PCC 7120	6,413,771	119	191	27	39	39	34
*Synechococcus elongatus* PCC 6301	2,696,255	142	113	10	41	7	28
*Synechococcus elongatus* PCC 7942	2,695,903	141	113	10	41	6	28
*Synechococcus elongatus* PCC 11801	2,691,022	139	115	10	41	7	29
*Synechococcus* sp. PCC 7002	3,008,047	153	163	22	48	11	33
*Synechococcus* sp. PCC 11901	3,081,514	152	163	22	47	11	33
*Synechococcus* sp. UTEX 2973	2,690,418	142	113	10	41	6	28
*Synechococcus* sp. WH 8102	2,434,428	173	86	7	49	4	28
*Synechocystis* sp. PCC 6803	3,569,561	161	174	23	51	12	32
*Thermosynechococcus elongatus* BP-1	2,593,857	176	126	13	42	10	27

**Table 5:** Genome-scale models currently available for different cyanobacterial strains.

Cyanobacteria	GSM Name	Reference
*Synechocystis* sp. PCC 6803	iSynCJ816	[230]
	imSyn716	[42]
*Synechococcus* sp. PCC 7942	iSyf715	[231]
	iJB785	[41]
	iJB792	[232]
*Synechococcus* sp. UTEX 2973	iSyu683	[233]
	imSyu593	[43]
*Synechococcus* sp. PCC 7002	iSpy708	[234]
	iSpy821	[235]
*Arthrospira platensis* NIES-39	n/a	[40]
*Nostoc* sp. PCC 7120	n/a	[236]
